# Silicon Application Modulates the Growth, Rhizosphere Soil Characteristics, and Bacterial Community Structure in Sugarcane

**DOI:** 10.3389/fpls.2021.710139

**Published:** 2021-08-20

**Authors:** Quanqing Deng, Taobing Yu, Zhen Zeng, Umair Ashraf, Qihan Shi, Suihua Huang, Tengxiang Lian, Jianwen Chen, Wardah Muzaffar, Wankuan Shen

**Affiliations:** ^1^College of Agriculture, South China Agricultural University, Guangzhou, China; ^2^Sugarcane Research Laboratory, South China Agricultural University, Guangzhou, China; ^3^The State Key Laboratory for Conservation and Utilization of Subtropical Agro-bioresources, South China Agricultural University, Guangzhou, Guangdong, China; ^4^Division of Science and Technology, Department of Botany, University of Education, Lahore, Pakistan; ^5^Scientific Observing and Experimental Station of Crop Cultivation in South China, Ministry of Agriculture, Guangzhou, China; ^6^Sugarcane Research Institute, Ayub Agricultural Research Institute, Faisalabad, Pakistan

**Keywords:** silicon, sugarcane, illumina miseq sequencing, soil enzymes activities, soil properties, bacterial community structure, association network analysis

## Abstract

Silicon (Si) deficiency, caused by acidic soil and rainy climate, is a major constraint for sugarcane production in southern China. Si application generally improves sugarcane growth; however, there are few studies on the relationships between enhanced plant growth, changes in rhizosphere soil, and bacterial communities. A field experiment was conducted to measure sugarcane agronomic traits, plant nutrient contents, rhizosphere soil enzyme activities and chemical properties, and the rhizosphere bacterial community diversity and structure of three predominant sugarcane varieties under two Si treatments, i.e., 0 and 200 kg of silicon dioxide (SiO_2_) ha^−1^ regarded as Si0 and Si200, respectively. Results showed that Si application substantially improved the sugarcane stalk fresh weight and Si, phosphorus (P), and potassium (K) contents comparing to Si0, and had an obvious impact on rhizosphere soil pH, available Si (ASi), available P (AP), available K (AK), total phosphorus (TP), and the activity of acid phosphatase. Furthermore, the relative abundances of *Proteobacteria* showed a remarkable increase in Si200, which may be the dominant group in sugarcane growth under Si application. Interestingly, the AP was noticed as a major factor that caused bacterial community structure differences between the two Si treatments according to canonical correspondence analysis (CCA). In addition, the association network analysis indicated that Si application enriched the rhizosphere bacterial network, which could be beneficial to sugarcane growth. Overall, appropriate Si application, i.e., 200 kg SiO_2_ ha^−1^ promoted sugarcane growth, changed rhizosphere soil enzyme activities and chemical properties, and bacterial community structures.

## Introduction

Silicon (Si), an ubiquitous and abundant (~28%) element in soil, is mostly insoluble and not readily available for plants (Liang et al., 2015; Mo et al., 2017). In general, Si has not been considered as an essential plant element; nevertheless, its roles in improving crop growth and inducing biotic and abiotic stress resistance in plants have been well-documented (Frayssinet et al., 2019; Lin et al., 2020). Sugarcane (*Saccharum* spp. hybrids) is globally recognized as a “sugar crop” and a renewable green energy crop (Chen et al., 2017; Liu X. et al., 2018). There are more than 100 cane-growing tropical countries that depend on the sugarcane industry for economic opportunities. For China, being the third largest sucrose producing country in the world, the sugarcane industry has had significant contributions for poverty alleviation (Li and Yang, 2014; Li W. F. et al., 2019).

Moreover, sugarcane is a moderate Si-accumulating and -responsive crop, with absorption rates higher than that for other mineral nutrients except for potassium (Meyer and Keeping, 2005; Liang et al., 2015); however, acidic soil and a rainy climate often result in Si deficiency, which affects the sugarcane growth in the dominant sugarcane regions in southern China (Liu et al., 2014; Liang et al., 2015; Keeping, 2017). Studies reported that exogenous Si application could significantly promote sugarcane growth and yield in Si-deficient soil (Keeping, 2017). Moreover, Si-mediated improvements in soil nutrient availability and plant nutrient absorption led to modulation in agronomic attributes of the sugarcane crop (Orndorff et al., 2018).

Generally, root–soil–microbe interaction has become an interesting research hotspot for environmentalists and in the areas of sustainable agriculture (Zhang et al., 2017). The diversity of soil microorganisms is critical for soil health; however, the higher density and diversity of microbial cells in the rhizosphere and their mutual interactions are especially complicated (Garbeva et al., 2004; Shi et al., 2016). Many researchers have evaluated that Si could actively participate in plant–microbe interactions (Fang et al., 2013; Zhou et al., 2018; Liu et al., 2019). Recently, it was found that exogenous Si could significantly affect soil microbial communities and the resistance of tomato against bacterial wilt (Liu et al., 2019). In addition, Si application alters soil physicochemical properties, which indirectly affects soil microbial communities (Karunakaran et al., 2013; Yang et al., 2018). Recently, Li M. et al. (2019) demonstrated that soil bacterial communities were largely affected by soil pH and available potassium after short-term Si application. Furthermore, the activity of soil enzymes was regulated by changing the structure and activity of soil microbes with Si application (Wang et al., 2013). Similarly, Zhou et al. (2018) showed that sodium silicate could change soil microbial communities to enhance the resistance of cucumber to Fusarium wilt.

The effects of Si on morphological growth of different crops were previously reported (Brindavathy et al., 2012); however, there are only a few available reports on the effects of Si-mediated changes in soil properties and soil microbial communities and their subsequent effects on crop growth. Therefore, the present study was performed to determine how Si application alteration the growth, soil enzymes activities, soil physiochemical properties, and rhizosphere bacterial diversity and structures in sugarcane, and the relationships among sugarcane, soil, and rhizobacteria under different Si levels. Additionally, the bacterial abundance and community structures were determined by Illumina MiSeq sequencing.

## Materials and Methods

### Experimental Site and Materials

A field experiment was conducted at the experimental farm (23°10″N, 113°21′E) of sugarcane, South China Agricultural University (SCAU) in Guangzhou, Guangdong Province, China. The properties of the field experimental soil (the upper 20 cm) comprised of 23.58 g kg^−1^ organic matter (OM), 0.85 g kg^−1^ of total nitrogen (TN), 0.87 g kg^−1^ of total phosphorus (TP), 20.13 g kg^−1^ of total potassium (TK), 72.63 mg kg^−1^ of alkali hydrolyzed nitrogen (AN), 36.26 mg kg^−1^ of effective phosphorus (AP), 51.43 mg kg^−1^ of available potassium (AK), 55.23 mg kg^−1^ of available Si (ASi), and 5.37 soil pH. The field soil was found to be Si-deficient, as the critical plant-available silicon dioxide (SiO_2_) content was 105–120 mg kg^−1^ (i.e., Si content was 49–56 mg kg^−1^) (Liang et al., 2015).

### Experimental Treatments

The experimental treatments were comprised of two basic Si fertilizer treatments, i.e., 0 and 200 kg of SiO_2_ ha^−1^ regarded as Si0 and Si200, respectively, and three sugarcane varieties (V), i.e., LC05-136 (VA), YT93-159 (VB), and ROC22 (VC), were obtained from the resource garden of the Sugarcane Breeding Base, SCAU, China. The stalks were carefully inspected to ensure that they were free of sugarcane diseases, and then cut into two-buds seed canes (Deng et al., 2020). The Si fertilizer was the beaded sodium metasilicate anhydrous (Na_2_SiO_3_, 45.5–47.5% SiO_2_; Haiwan Chemical Co., Ltd., Qingdao, China), which was used as a base fertilizer. A two-factor (i.e., sugarcane varieties and Si application) factorial in a randomized block design was used in the experiments with three blocks. Each block contained six random plots, with the length and width of each plot being 2 and 3 m, respectively (i.e., row spacing was 1 m, with 3 rows in total), and a 1-m gap between each.

The 36 two-buds seed canes (i.e., 120,000 buds ha^−1^) were sown per subplot on January 1, 2019. Before planting, the seed canes were disinfected with 5% carbendazim for 15 min. The basal fertilizer (N:P:K) was applied with 80:240:230 kg ha^−1^ in the form of urea, potassium chloride, and calcium superphosphate in all treatments, respectively. All other crop management practices were managed by following the guidelines recommended by the province.

### Rhizosphere Soil and Plant Sampling

Rhizosphere soil sampling from 15 random plants of each treatment (triplicate, and 5 plants per plot) were done *via* shaking the root for 2 min into a polyethylene bag and mixing thoroughly (Lian et al., 2019). The sampling time was done at 15:00–17:00 during the early elongation stage (i.e., June 8, 2019). Five random plants from each plot were uprooted manually and washed with tap water. Then, the fresh weight per stalk, plant height, and stalk diameter of all plants were measured and immediately divided into leaves, stalks, and root after soil sampling. The plant height was recorded from the stalk base to the first visible dewlap leaf. The stalk diameter was investigated at one-third of the plant height (from the basis to the top). The plant organs were dried in an oven at 85°C after washing with distilled water, ground, and passed through a 100-mesh sieve to measure the contents of the plant nutritional elements (i.e., plant TN, TP, TK, and Si). In addition, the soil samples were mixed and divided into three portions: one portion was frozen with liquid nitrogen and stored at −80°C for DNA extraction; one portion was stored at −20°C for determination of soil enzymes; the rest were dried at room temperature and passed through a 100-mesh sieve for determination of soil chemical properties.

### Plant and Soil Chemical Analysis

The dried plant (0.20 g) and air-dried soil (0.50 g) samples were digested with sulfuric acid (H_2_SO_4_):hydrogen peroxide (H_2_O_2_) (5:2 ratio) and H_2_SO_4_:perchloric acid (HClO_4_) (5:1 ratio), respectively. The resulting digestion solutions and the methods described by Lu (1999) were used to determine TN, TP, and TK. The total Si content was measured according to the high-temperature alkaline melting method (Fox et al., 1969; Dai et al., 2005). On the other hand, the soil ASi content was quantified using the citric acid extraction method (Liu et al., 2017). The total Si and soil ASi are elemental Si, not SiO_2_. The soil pH was estimated in a soil–water suspension (1:5 w/v) *via* a pH analyzer (DZB-712, INESA Scientific Instrument Co., Ltd., Shanghai, China). The soil OM content was determined according to Beaudoin (2003). The TN contents of the plants and soil were evaluated by an automatic Kjeldahl analyzer (K1100, Hanon Instruments Co., Ltd., Jinan, China). The soil AN content was measured by the alkali N-proliferation method. The TP and soil AP contents were evaluated by the molybdenum blue colorimetric method. The TK and soil AK contents were analyzed using the Atomic Absorption Spectrophotometer (AA-6300C, Shimadzu, Japan). Soil acid phosphatase (SAP), catalase (SCAT), and invertase (SI) activities were determined using the methods of Guan et al. (1986).

### Illumina MiSeq Sequencing and Data Processing

The total DNA was extracted from 0.5 g of each soil sample using the FastDNA Spin Kit for Soil (MP Biomedical, Santa Ana, CA, USA) according to the instructions given by the manufacturer. The integrity of the extracted DNA was detected by electrophoresing on a 1% (w/v) agarose gel; the concentration and purity were evaluated based on 260/280 and 260/230 nm absorbance ratios obtained using an ultra-micro UV spectrophotometer NanoDrop ND-1000 (Thermo Fischer Scientific, Wilmington, DC, USA).

For high-throughput sequencing, the primers 515F (5′-GTGCCAGCMGCCGCGGTAA-3′) and 907R (5′-CCGTCAATTCMTTTRAGTTT-3′) with 8-nt unique barcodes were performed to amplify the V4–V5 hypervariable region of the bacterial 16S rRNA gene (Zhou et al., 2011). To determine the rhizosphere soil bacterial community composition and diversity in each soil sample, equivalent amounts of purified amplified products were pooled and paired-end sequenced with the Illumina MiSeq platform according to standard protocols at MAGIGENE Biotech Co., Ltd. (Guangzhou, China). All sequences were deposited into the GenBank Sequence Read Archive with an accession: PRJNA656199.

After sequencing, the raw fastq files were processed using QIIME (version 1.17, http://qiime.org/). Generally, all sequence reads were matched to each sample according to the barcodes. For further analysis, the amplicons with sequences shorter than 200 bp and average base quality score <25 were removed. The chimera of trimmed sequences with lengths between 245 and 258 bp was removed using U-Chime (Edgar et al., 2011). The processed sequences were classified and analyzed by the Ribosomal Database Project (RDP) database (http://rdp.cme.msu.edu/) using the RDP classifier. The sequences were clustered into operational taxonomic units (OTUs) at 97% sequence similarity with UPARSE (Edgar, 2013). Relative abundance (%), Chao1 richness, and Shannon's diversity index were estimated using the method described by Liu S. Q. et al. (2018).

### Statistical Analysis

The program R version 4.0.0 for Windows with the “vegan” package was applied to perform non-metric multidimensional scaling (NMDS), canonical correspondence analysis (CCA), significance tests (ADONIS test and mantel test), and association network analyses (Lian et al., 2019). The differences in genera between Si0 and Si200 were analyzed using STAMP (version 2.1.3) with 95% confidence intervals (Parks and Beiko, 2010). A volcano plot was utilized for discriminating OTUs (i.e., enriched and depleted OTUs) that significantly correlated with community separation between genotypes or treatments. An UpSet plot (https://www.omicstudio.cn) was performed to reveal which of the enriched OTUs were shared between the two Si treatments. The Statistix version 8.0 for Windows (Analystical, Tallahassee, FL, USA) was applied to perform ANOVA, and all pairwise comparisons were used to analyze the differences among treatments by using the least significant difference (LSD) and Sidak–Holm test at a 5% probability level. The SPSS version 21 software (IBM Corp., Armonk, NY, USA) was used for the paired sample *t*-test with 95% confidence intervals and the Pearson correlation test between OTUs and Si treatments. The multivariate analysis, i.e., heatmap, PatternsHunter, principal component analysis (PCA), partial least squares-discriminant analysis (PLS-DA), and score plot, was performed by the MetaboAnalyst software (http://www.metaboanalyst.ca) (Mo et al., 2019).

## Results

### Sugarcane Growth Responses

Except for Si and N in stalk and root, ANOVA of the interactions with the treatments (Table 1, Supplementary Table 1) showed that the agronomic traits and the nutritional elements were significantly affected by variety (V). The analysis also showed that treatment (T) had a significant influence on the agronomic traits, Si in stalk and root, N in stalk and root, and K in all plant organs. The interaction between V and T (V × T) had a significant effect on the above-mentioned indices except for stalk N contents. For agronomic traits, compared with Si0, Si200 notably (*P* < 0.05) improved the fresh weight of varieties A, B, and C by 7.96, 10, and 17.24%, respectively, and plant height significantly increased by 6.51, 4.07, and 2.91%, respectively. Stalk diameter, on the other hand, decreased significantly in the VB and VC varieties by 1.56 and 6.16%, respectively (Table 1). Likewise, the Si contents in plants (leaf, stalk, and root) were significantly enhanced under Si200 for all varieties except for the leaves in VC and the stalks in VB, while the Si content was recorded as root > leaf > stalk at the early elongation stage (Supplementary Table 1). Furthermore, the N contents in leaves and roots were significantly enhanced under Si200 in all varieties except for the leaves in VB, the stalks in all varieties, and the roots in VB compared with the contents of plants under Si0; for the P and K content, significant differences were shown in all parts of the plants for all varieties except for the leaves in VB, the stalks in VC, the stalks in VB, and the roots in VC.

**Table 1:** Growth parameters of the three sugarcane varieties under the two silicon (Si) treatments.

**V**	**T**	**Fresh weight**	**Plant height**	**Stalk diameter**
		**(kg plant^**−1**^)**	**(cm plant^**−1**^)**	**(cm plant^**−1**^)**
A	Si0	1.13 ± 0.01b	125.00 ± 2.19b	3.04 ± 0.05c
	Si200	1.22 ± 0.02a	133.14 ± 1.82a	3.06 ± 0.03c
B	Si0	0.80 ± 0.01e	108.77 ± 2.33f	3.26 ± 0.06a
	Si200	0.88 ± 0.02d	113.20 ± 2.64e	3.21 ± 0.03b
C	Si0	0.87 ± 0.04d	117.23 ± 1.87d	2.93 ± 0.05d
	Si200	1.02 ± 0.01c	120.64 ± 2.36c	2.76 ± 0.05e
ANOVA	V	**	**	**
	T	*	*	*
	V × T	**	**	**

Different lowercase letters within a column indicate a significant difference under each treatment according to the least significant difference (LSD) (0.05). The error term is SE. ns, not significant at the 0.05 probability level; * and **, significant at the 0.05 and 0.01 probability levels, respectively. V, variety; T, treatment.

### Rhizosphere Soil Physiochemical Properties and Enzymes

Variety had significant effects on soil pH, ASi, SOM, TN, and SAP (Table 2, Supplementary Table 2), whereas T significantly affected the soil pH, ASi, AP, AK, SOM, TN, TP, TK, and SAP. The interaction between V and T indicated significant effects on pH, ASi, AN, AP, AK, TP, SOM, TN, TP, TK, and SAP. Regarding soil physiochemical properties, compared with Si0, the SAP activities were reduced by 11.5, 11.71, and 11.78% in variety A, B, and C, respectively, whereas the activities of SCAT and SI were not significantly different (Table 2). In addition, as shown in Supplementary Table 2, the soil pH was significantly improved (*P* < 0.05) by 5.4, 3.05, and 3.13% in variety A, B, and C, respectively, while the ASi content was significantly (*P* < 0.01) increased by 15.17, 26.44, and 26.52% in A, B, and C, respectively. The AP contents were significantly increased by 22.73, 9.69, and 29.45% in A, B, and C, respectively, whereas the AK contents were significantly enhanced by 39.93, 24.34, and 8.62%. The AN contents were increased by 2.06% in VA only. The SOM contents were increased by 12.17% in VC only. Likewise, the TN contents were increased by 10.71% in VA only, whereas the TP contents were enhanced by 5.07, 5.09, and 2.34% in A, B, and C, respectively, and the TK contents were increased by 2.57% in VC only.

**Table 2:** Soil enzyme activities of the three sugarcane varieties under the two silicon (Si) treatments.

**V**	**T**	**Catalase**	**Acid phosphatase**	**Invertase**
		**(mL g^**−1**^ FW)**	**(mg g^**−1**^ 24 h^**−1**^ FW)**	**(mg g^**−1**^ 24 h^**−1**^ FW)**
A	Si0	0.088 ± 0.001a	0.788 ± 0.003d	1.041 ± 0.006a
	Si200	0.087 ± 0.001a	0.697 ± 0.003f	0.922 ± 0.076a
B	Si0	0.088 ± 0.001a	0.934 ± 0.006a	1.063 ± 0.095a
	Si200	0.089 ± 0.001a	0.824 ± 0.008c	1.147 ± 0.080a
C	Si0	0.088 ± 0.002a	0.854 ± 0.006b	1.021 ± 0.004a
	Si200	0.088 ± 0.001a	0.754 ± 0.002e	0.928 ± 0.065a
ANOVA	V	ns	**	ns
	T	ns	**	ns
	V × T	ns	**	ns

Different lowercase letters within a column indicate a significant difference under each treatment according to the least significant difference (LSD) (0.05). The error term is SE. ns, not significant at the 0.05 probability level; * and **, significant at the 0.05 and 0.01 probability levels, respectively. V, variety; T, treatment.

### Correlation Between Fresh Weight and the Investigated Parameters

**Figure 1:**
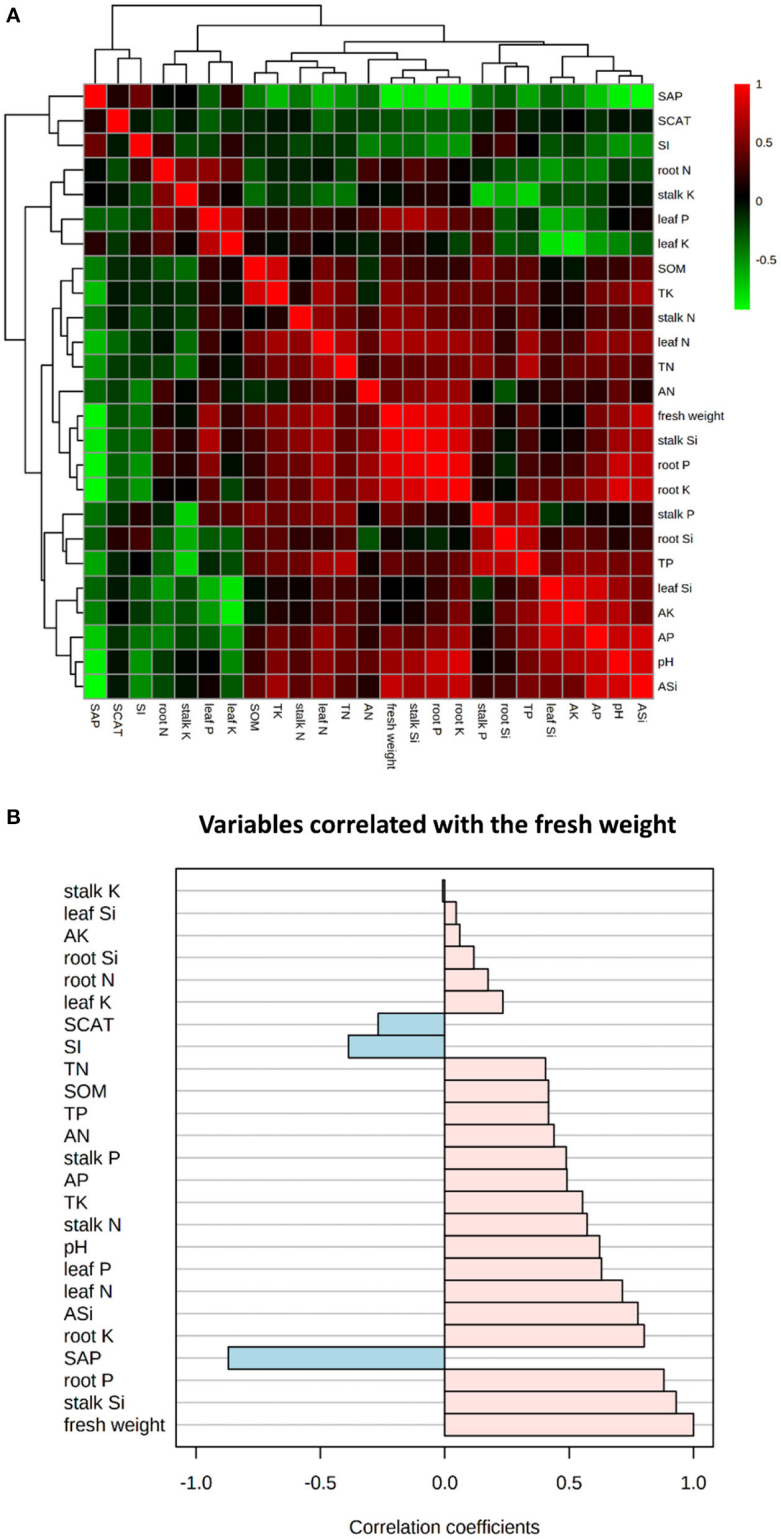
The heatmap **(A)** for fresh weight and the investigated parameters, and the top 25 parameters correlated with fresh weight **(B)**. Red and green grids represent positive and negative correlation, respectively; the brighter the color, the stronger the correlation, and vice versa. ASi, available Si; AN, alkali hydrolyzed nitrogen; AP, available phosphorus; AK, available potassium; TN, total nitrogen; TP, total phosphorus; TK, total potassium; SOM, soil organic matter; SAP, soil acid phosphatase; SCAT, soil catalase; SI, soil invertase.

Significant (*P* < 0.05) positive correlations between fresh weight and several of the investigated parameters (i.e., leaf P, TK, stalk N, leaf N, stalk Si, root P, root K, AP, pH, ASi) were noticed, while significant (*P* < 0.05) negative correlation between fresh weight and SAP was noted (Figure 1A). To further find the possible relationships between fresh weight and the investigated parameters, all the investigated parameters that strongly correlated with fresh weight were sorted (Figure 1B), which then suggested that physiological attributes like stalk Si, root P, SAP, root K, ASi, leaf N, pH, stalk N, and TK were strongly related to cane fresh weight.

### PCA and PLS-DA

**Figure 2:**
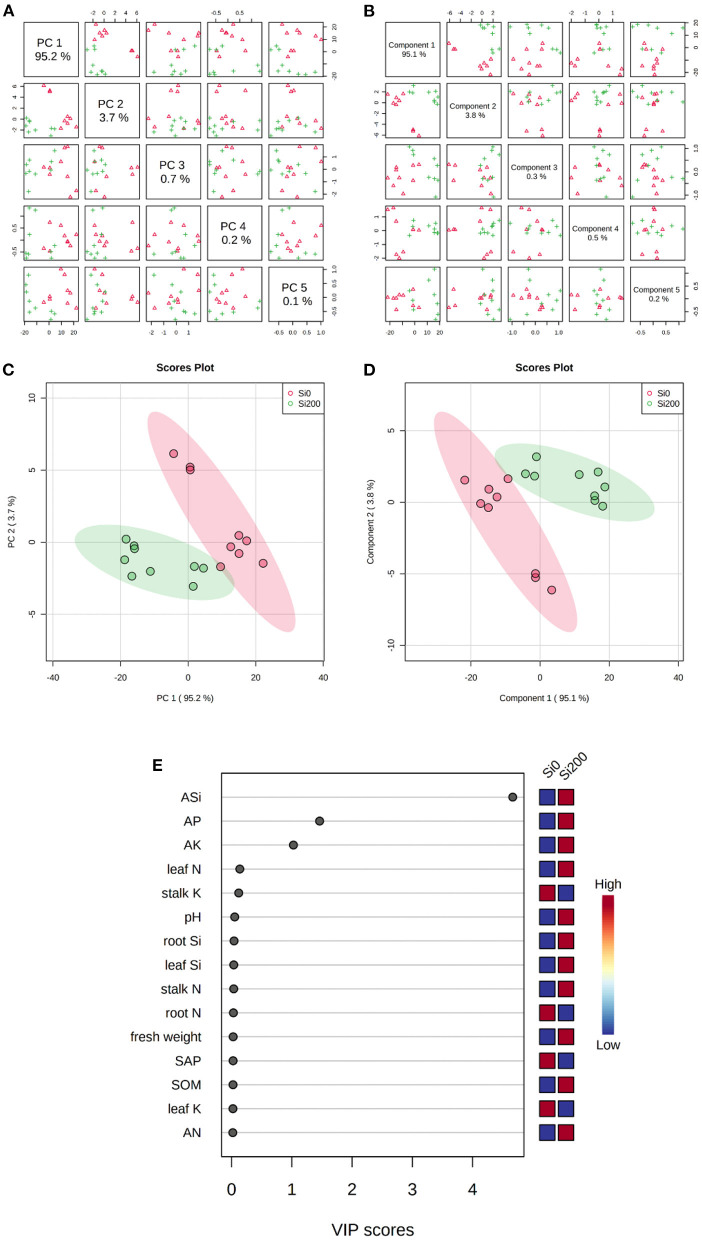
Principal component analysis (PCA) **(A,C)** and partial least squares-discriminant analysis (PLS-DA) **(B,D)** of the investigated parameters, and the variable importance in projection (VIP) to component 1 of the PLS-DA for the two Si treatments **(E)**.

The principal component analysis and partial least squares-discriminant analysis (PLS-DA) were performed to visualize of the consistency of the investigated parameters and identify the key parameters under the two Si treatments. The PCA (Figures 2A,C) of the investigated parameters indicated that the percent of variance for PC1, PC2, PC3, PC4, and PC5 was 95, 3.7, 0.7, 0.2, and 0.1%, respectively. The PLS-DA (Figures 2B,D) revealed five components, i.e., component 1 (95%), component 2 (3.8%), component 3 (0.3%), component 4 (0.5%), and component 5 (0.2%). It can be seen from Figure 2C and d that the two Si treatments could be completely separated by the visual analysis. Further analysis of the variable importance in projection (VIP) according to component 1 of the PLS-DA was conducted to quantify the contribution of each parameter to the separation of the two Si treatments. Therefore, the ASi, AP, and AK were the core parameters (VIP score > 1) that were associated with two Si treatments based on component 1 (95%), as established by the PLS-DA (Figure 2E).

### Rhizosphere Soil Diversity Indices

The 16S rRNA sequencing of 18 soil samples generated 6,308,443 high-quality sequences and 56,701–108,280 bacterial sequences at a 97% similarity level. Data homogenization (i.e., reads number = 50,859) was performed at the reads level, and then the ANOVA and all pairwise comparisons were applied for the diversity indices (OTUs, Chao1, Shannon_e) of the two Si treatments of the three varieties. However, V, T, and V × T had no significant effect on the diversity indices (Supplementary Table 3). The coverage index of the soil samples library was more than 98.07%, indicating that the sequencing results could reflect the real situation of the bacterial community.

### Rhizosphere Soil Bacterial Taxonomic Classification and Relative Abundance

**Figure 3:**
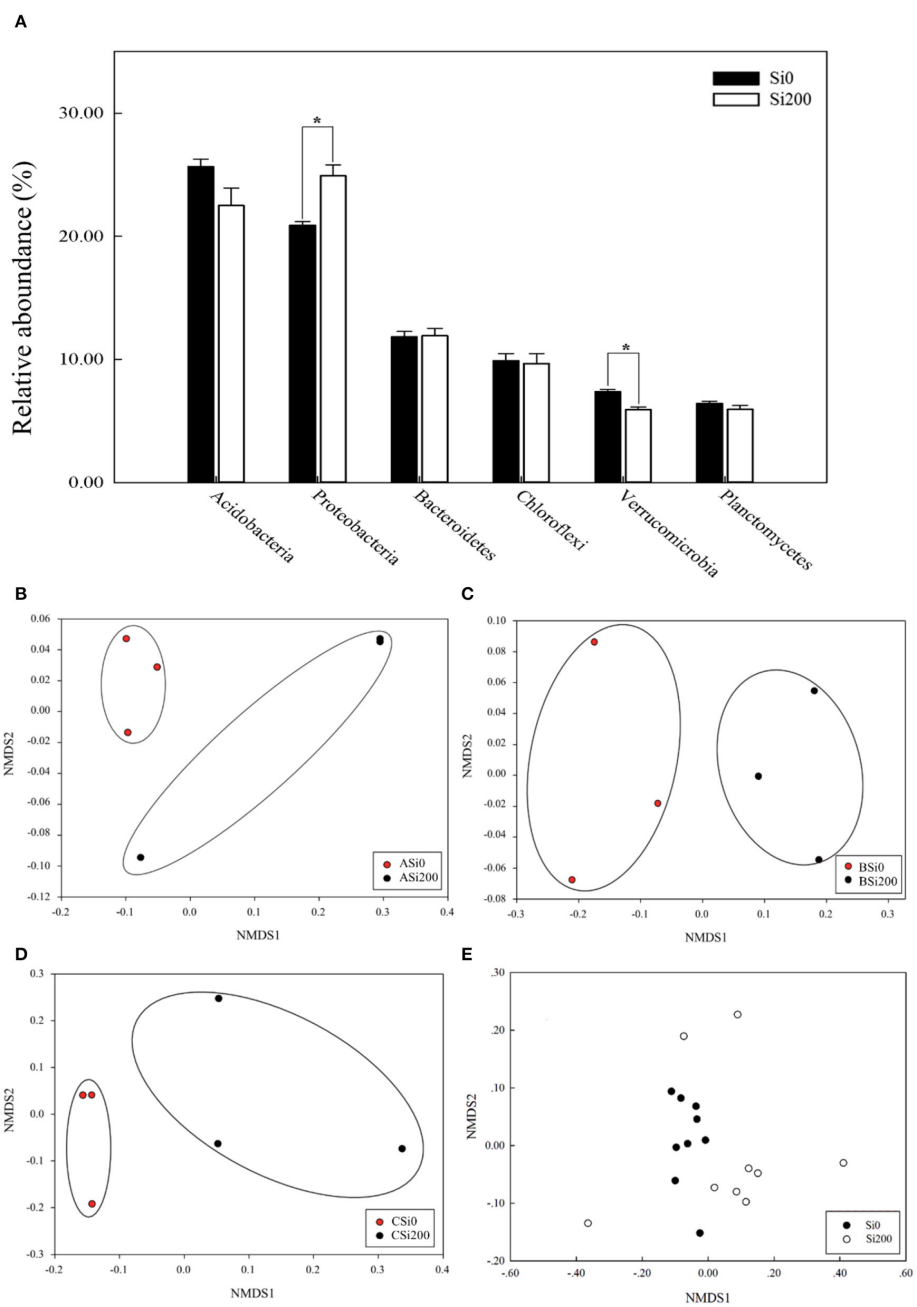
The relative abundances of the five most enriched bacterial phyla in the three sugarcane varieties under the two silicon (Si) treatments **(A)**. Non-metric multidimensional scaling (NMDS) based on the Bray–Curtis dissimilarity matrix in the two Si treatments of VA **(B)**, VB **(C)**, VC **(D)**, and all soil samples **(E)**. *,Significant at the 0.05 probability level.

The dominant (the relative abundance >5%) bacterial phyla were *Acidobacteria, Proteobacteria, Bacteroidetes, Chloroflexi, Verrucomicrobia*, and *Planctomycetes* with relative abundance varying from 19 to 32.39%, 18.01 to 32.62%, 7.51 to 18.81%, 5.71 to 20.1%, 4.32 to 8.7%, and 2.86 to 8.13% in all soil samples, respectively (Figure 3A). Moreover, compared with Si0, the relative abundance of *Proteobacteria* under Si200 was significantly (*P* < 0.05) improved by 19.23%, while that of *Verrucomicrobia* was significantly decreased by 24.6%. In general, the phyla responded to Si treatments and varied between varieties, which is consistent with the results of the NMDS (Figures 3B–E). Further analysis, i.e., ADONIS test (*P* < 0.05), was further performed to test the relative abundances under the two Si treatments at different taxon levels. There was a significant (*P* < 0.05) difference at the order, family, genus, and species levels but no significant differences were noted at the phylum and class levels (Table 3). These results indicated significant differences in bacterial community structures.

**Table 3:** The significance analysis of community structure differences (using the ADONIS test) between groups under the two silicon (Si) treatments with different taxon levels.

**Taxon**	**F**	**R^2^**	***P***
Phylum	2.09	0.115	0.121
Class	1.94	0.108	0.107
Order	3.02	0.159	**0.015**
Family	3.15	0.164	**0.007**
Genus	2.93	0.155	**0.002**
Species	5.18	0.245	**0.002**
OTUs	3.69	0.188	**0.003**

*R^2^, the percentage of variation explained by treatments. P, differential clustering assessed by ADONIS test. Bold font indicates significance at the 0.05 probability level*.

**Figure 4:**
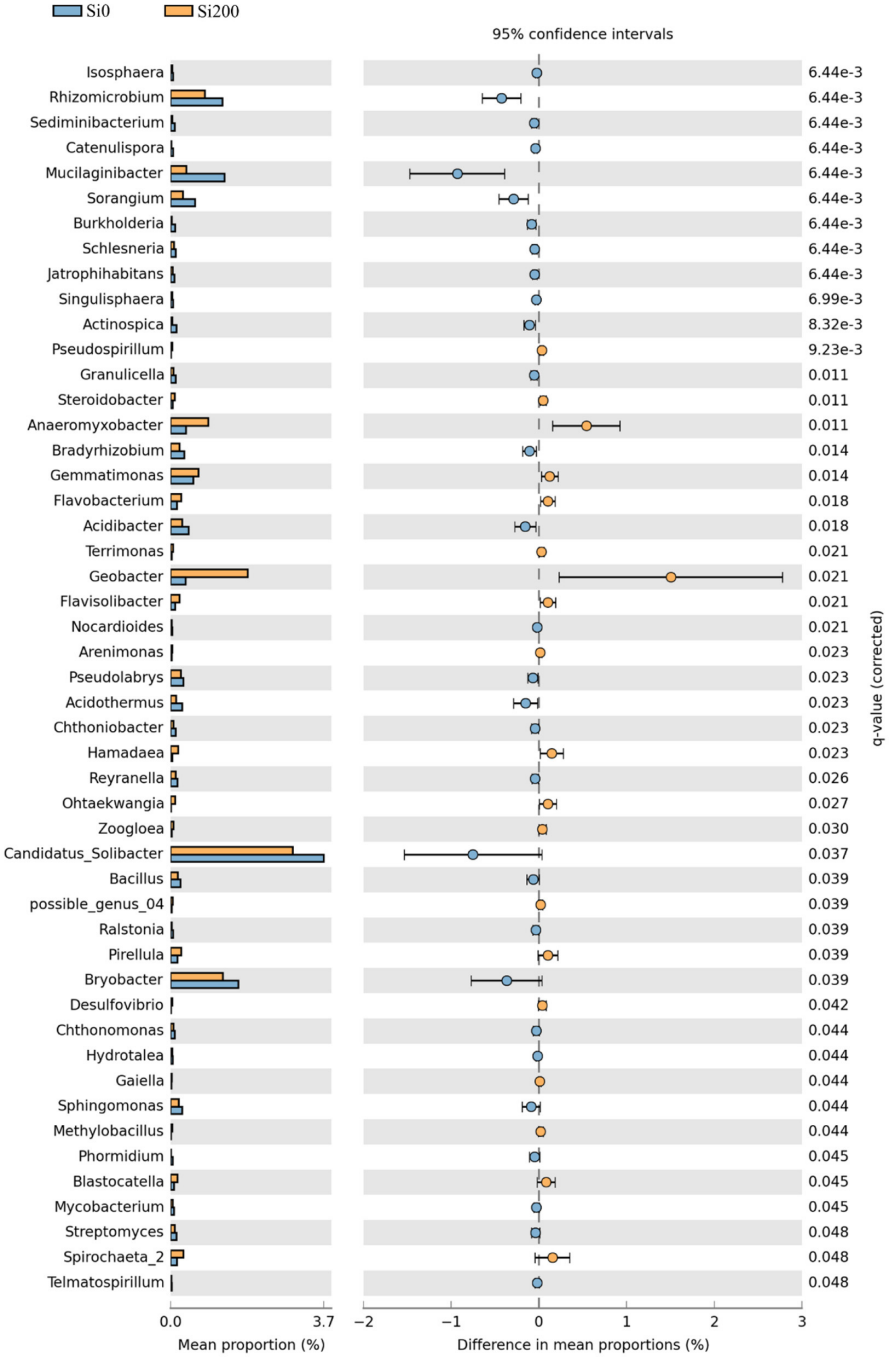
Topological properties of bacterial networks obtained from the rhizospheres of the two silicon (Si) treatments. Corrected *P*-values were calculated using the Story false discovery rate approach (*P* < 0.05).

The differences of the top 150 genera between the two Si treatments in the three varieties were analyzed using 95% confidence intervals. There were 30 significant genera at Si0 and 19 at Si200 (Figure 4). Compared with Si0, there were 2 (*Pirellula* and *Gemmata*), 14 (*Geobacter, Dechloromonas, Pirellula*, etc.), and 4 (*Pseudospirillum, Terrimonas, Zoogloea*, and *Steroidobacter*) genera with higher relative abundances in Si200 treatment for VA, VB, and VC, respectively (Supplementary Figure 1).

**Figure 5:**
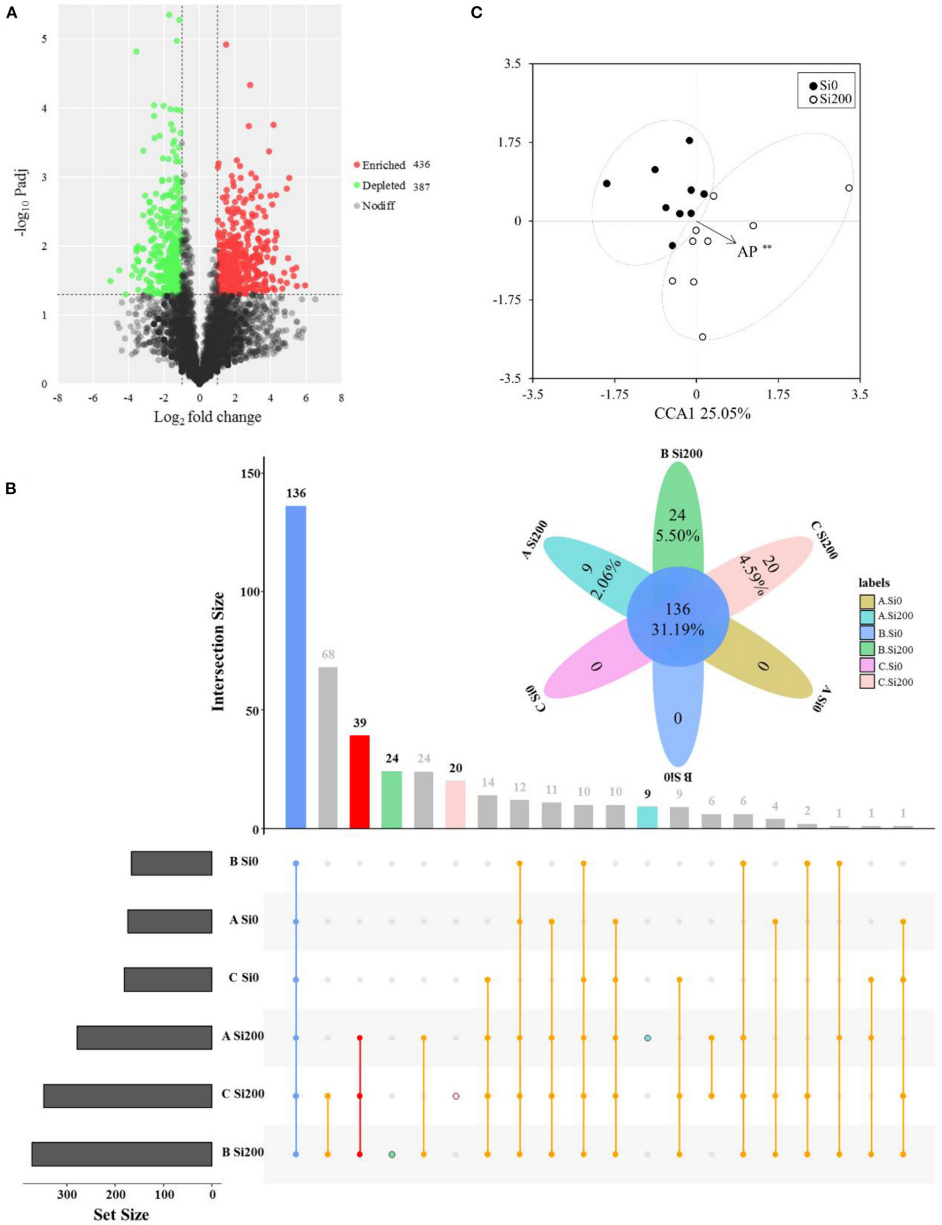
Enrichment and depletion of operational taxonomic units (OTUs) of the three varieties **(A)** included in the treatment with 200 kg of silicon dioxide (SiO_2_) ha^−1^ (Si200) compared with the treatment with 0 kg of SiO_2_ ha^−1^ (Si0) as determined by differential abundance analysis. Each point represents an individual OTU, and the position along the y-axis represents the abundance fold-change compared with Si0 **(A)**. UpSet plot showing the number of OTUs that are unique or shared between the three sugarcane varieties under three two silicon (Si) treatments **(B)**. Blue column, the common OTUs for each treatment; red column, the common OTUs for Si200 treatments; light blue, green, orange column, the unique OTUs for ASi200, BSi200, CSi200, respectively **(B)**. Canonical correspondence analysis (CCA) based on the bacterial community compositions of three sugarcane varieties samples **(C)** under the two Si treatments. *,Significant at the 0.05 probability level.

The volcano plot showed that the rhizosphere soil of the Si200 treatment had higher enriched (436) OTUs as compared with Si0 (Figure 5A). The numbers of enriched OTUs were 54, 301, and 75, respectively, and the numbers of depleted OTUs were 118, 183, and 164 for VA, VB, and VC under the two Si treatments, respectively (Supplementary Figures 2a–c). As shown in Figure 5B, 39 OTUs were only enriched in Si200 for the 3 varieties, and the relative abundances of these showed significant correlations with Si treatments including OTU975 (*Bryobacter*), OTU3504 (*Flavobacterium*), OTU3634 (*Cytophaga*), and OTU4956 (*Flavisolibacter*) (Figure 5B, Supplementary Table 4). Further, there were 9, 24, and 20 OTUs that were only enriched in Si200 for VA, VB, and VC, respectively (Figure 5B).

### Rhizosphere Soil Properties and Community Structures

The CCA was performed to establish the links of the rhizosphere soil physiochemical properties to the compositions of bacterial communities in the VA (Supplementary Figure 3a), VB (Supplementary Figure 3b), and VC (Supplementary Figure 3c) varieties and the two Si treatments (Figure 5C). The Mantel test was further applied to find the Spearman's correlations between the rhizosphere soil properties and the bacterial community structures based on the Bray–Curtis distance. The results displayed that bacterial community structure was significantly (*P* < 0.05) correlated with ASi, AP, and SI for VA under two Si treatments, pH and SAP significantly (*P* < 0.01) for VB, and AP and SI significantly (*P* < 0.05) for VC (Figure 5C, Supplementary Figures 3a–c, Supplementary Table 5). Only AP significantly correlated with bacterial community structures between Si0 and Si200 for the three varieties (Figure 5C, Supplementary Table 5).

### Association Network Analysis of Rhizosphere Soil Bacterial Community Structures

**Figure 6:**
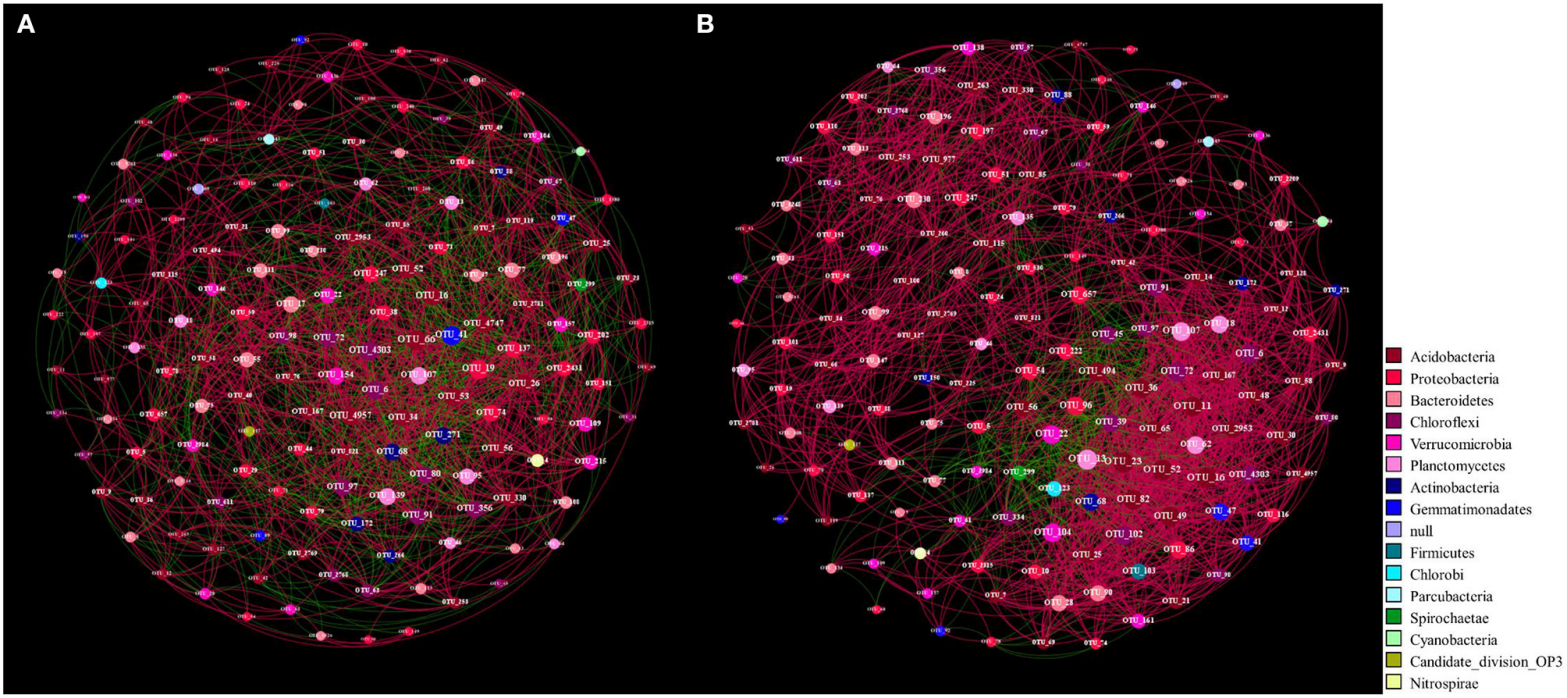
Networks of operational taxonomic units (OTUs) (relative abundance >0.1%) in the rhizospheres of the 0-kg silicon dioxide (SiO_2_) ha^−1^ (Si0) **(A)** and 200-kg SiO_2_ ha^−1^ (Si200) application treatment **(B)** included in the three sugarcane varieties based on correlation analysis. A connection stands for a strong (Spearman's *r* > 0.6) and significant (*P* < 0.05) correlation. The networks are colored by phylum. For each panel, the size of each node is proportional to the number of connections (i.e., degree). A red edge indicates a positive interaction between two individual nodes, and a green edge indicates a negative interaction.

Further association network analysis was performed to explore the interaction of the rhizosphere bacteria of the two Si treatments based on strong (Spearman's *r* > 0.6) and significant (*P* < 0.05) correlations (Figure 6, Table 4). Overall, the number of edges, the number of positive correlations, the graph density, the *avgCC*, and the *avgK* of Si200 increased as compared with Si0. These results suggest that the complexity of the network increased in Si200; however, the average path length (APL) and modularity (M) of Si200 decreased (Table 4).

**Table 4:** Topological properties of the bacterial networks obtained from the rhizospheres of the two silicon (Si) treatments.

**Network metrics**	**Si0**	**Si200**
Number of nodes	162	162
Number of edges	1,784	2,057
Number of positive correlations	1,038 (58.18%)	1,674 (81.38%)
Number of negative correlations	746 (41.82%)	383 (18.62%)
Average path length (APL)	2.43	2.413
Graph density	0.137	0.158
Network diameter	5	5
Average clustering coefficient (*avgCC*)	0.561	0.601
Average degree (*avgK*)	22.025	25.395
Average weighted degree	3.496	13.953
Number of modules	1	1
Modularity (M)	4.909	0.73

## Discussion

The present study explains the effects of Si application on the rhizosphere bacteria of various sugarcane genotypes and illuminates the relationships between sugarcane, rhizosphere soil, and rhizobacteria. Our results revealed that Si application substantially improved sugarcane growth and changed rhizosphere soil properties and bacterial diversities and compositions. These results are applicable and suggest that appropriate Si application could contribute to sugarcane growth by driving different soil enzyme activities and altering soil chemical properties and bacterial community structures.

### Effect of Si on Sugarcane Growth, Soil Enzyme Activity, and Properties

Previous studies reported that Si could promote plant growth and affect rhizosphere soil enzyme activities and chemical properties (Zhou et al., 2011; Wang et al., 2013; Shen et al., 2018), but the relationships among these indices under Si application were not explored previously. Compared to Si0, Si application markedly increased the sugarcane fresh weight and Si, P, and K contents, but had no significant effect on stalk diameter and N (Table 1 and Supplementary Table 1), which corroborates the findings of Shen et al. (2018) that Si application could improve sugarcane growth. Our results showed that sugarcane, as a medium-silicophilic crop, has a significant positive correlation between its Si content of each organ and the fresh weight under appropriate Si application (Figure 1A), which is consistent with Mo et al. (2017) who found that Si application significantly increased the Si contents in the leaves of fragrant rice. The effects of Si application on sugarcane absorption and utilization of N, P, and K shows that Si could rebalance mineral nutrients and, indeed, promote plant growth (Liang et al., 2015).

Moreover, the Si supplement for sugarcane had a significant impact on rhizosphere soil pH, ASi, AP, AK, TP, and the activity of acid phosphatase (Table 2, Supplementary Table 2, and Figure 1A). Generally, soil enzyme activities and chemical properties are important indicators to predict and evaluate soil fertility (Caldwell, 2005). The soil pH was effectively improved by the applied Si fertilizer (i.e., alkaline sodium silicate) (Tavakkoli et al., 2011), which enhanced soil P and K availability (Mathew et al., 2017; Greger et al., 2018). The soil AP of acid soils is usually low due to its poor solubility, sorption, and slow diffusion (Liang et al., 2015). In this study, the enhancement of soil AP might have two explanations: competitive exchange of Si and P and increased soil pH (Figure 1A) (Smyth and Sanchez, 1980; Lee et al., 2004). Furthermore, SAP is one of the enzymes that regulate phosphorus availability in soil (Moscatelli et al., 2005). Compared with Si0, the activity of SAP notably decreased and had a negative correlation with the soil chemical properties under Si application (Table 2, Figure 1A). Previous reports indicated that an inverse relationship exists between phosphorus and SAP (Moscatelli et al., 2005; Nannipieri et al., 2010), whereas PLS-DA showed that the soil ASi, AP, and AK were significantly altered with Si application (Figure 2). Therefore, the present study also suggests that some beneficial effects of Si on sugarcane growth are indirect and related to Si-mediated changes in plant nutrient absorption, soil enzyme activity, and chemical properties.

### Effect of Si on Bacterial Community Diversity and Structure

Microbial community diversity is a major component of soil health (Garbeva et al., 2004). Our results indicated that the Chao 1 richness and Shannon diversity of the rhizosphere soil bacterial community was not significantly different across the three varieties under the Si treatments (Supplementary Table 3), which indicated that Si200 did not change the number of bacterial species (Li M. et al., 2019). One possibility is that the rhizospheric microbial community was influenced by various factors including the soil type, plant species, and plant growth stages (Wieland et al., 2001). Notably, the relative abundances of *Proteobacteria* showed a remarkable increase in Si200 (Figure 3A), which is similar to the findings of Solanki et al. (2020), which found that *Proteobacteria* were the dominant groups in sugarcane cultivation. Furthermore, Cheng et al. (2020) showed that *Proteobacteria* were sensitive to P fertilization, and their relative abundance was remarkably richer in P-treated soil. In addition, the results of the NMDS indicated that Si regulated the soil bacterial community structure between the treatments (Table 3, Figures 3B–E). These results are similar to those of Li M. et al. (2019), who found that Si application significantly regulated the impact of soil microorganism structures caused by ginseng black spot, though there was no significant difference in bacterial diversity. Overall, the results showed that Si application rather than sugarcane variety could markedly affect the rhizosphere soil bacterial community structure.

Interestingly, the genus- and OTUs-level significant differences were found between the two Si treatments, and Si enhanced the relative abundances of several microbial genera with plant growth-promoting potentials (Table 3, Figures 4, 5). Generally, plant growth-promoting rhizobacteria (PGPR) colonize within plant roots, and, therefore, modulate plant growth directly or indirectly (Vacheron et al., 2013; Ramakrishna et al., 2020). In this study, the differential OTU abundance analysis was used in all treatments and focused on the high relative abundance OTUs affiliated with *Bacteroidetes, Proteobacteria*, and *Acidobacteria*, which were enriched in Si200 (Figure 5, Supplementary Figure 3, Supplementary Table 4). These results are in line with those of Zhang et al. (2019), who found that the bacteria belonging to the *Bacteroidetes* and *Proteobacteria* were enriched in fallow soil in a sugarcane cropping system (Zhang et al., 2019). Most importantly, some OTUs enriched in Si200 exhibited higher positive correlations with Si application and were present in higher abundance in Si200 than Si0 (Figure 5C, Supplementary Table 4). This finding demonstrated that Si may activate PGPR to promote sugarcane growth. Furthermore, OTU975 (*Bryobacter*), OTU3504 (*Flavobacterium*), OTU3634 (*Cytophaga*), and OTU4956 (*Flavisolibacter*) indicated higher relative abundances in Si200 than Si0 (Supplementary Table 4). The higher abundances of *Flavobacterium* may increase sugarcane growth, improve soil AP and AK, inhibit plant pathogens, and produce large quantities of indole-3-acetic acid (IAA) for the promotion of plant growth (Pishchik et al., 2002; Meena et al., 2015; Vijayabharathi et al., 2016; Cardoso et al., 2018). Many studies reported that *Bryobacter* are PGPR, and could use organic acids, polysaccharides, and various sugars to participate in the biogeochemical carbon cycle (Dedysh et al., 2017; Liu et al., 2019). Aballay et al. (2012) inferred that *Cytophaga* could effectively protect grapevine roots from damage by the nematode *Xiphinema index*, which was also confirmed by Hu et al. (2019) for its ability of cellulose degradation. Nonetheless, *Flavisolibacter* were not recognized as PGPR, although they might still be associated with plant growth (Yang et al., 2017). Therefore, there is a need for future studies focusing on the response of these genera to different Si concentrations and their functional significance in response to sugarcane growth.

Furthermore, the CCA has been widely applied to illustrate the relationship between microbial community structures and environmental factors (Zhang et al., 2016). In this study, the results of the CCA based on the Si treatment samples showed that the rhizosphere bacterial community structures in the two treatments had a strong correlation with specific soil enzyme activities and chemical properties (Figure 5C, Supplementary Figure 3, Supplementary Table 5). These results speculated that Si application significantly altered these soil parameters, which, in turn, affected the rhizosphere bacterial community structures. Meanwhile, AP was the only macronutrient that exhibited a positive correlation with the bacterial community in Si200. These findings agree with Zhang et al. (2016), who reported that AP was significantly correlated with rhizosphere bacterial diversity. Furthermore, this study found that Si application significantly increased the abundance of *Proteobacteria*, which may be related to the increase of AP in the rhizosphere soil caused by Si. Cheng et al. (2020) showed that *Proteobacteria* were sensitive to P fertilization, and their relative abundance were remarkably richer in P-treated soil.

The present study also found lower abundances of *Bacillus* and *Burkholderia* in Si200, which both belong to inorganic phosphate solubilizing bacteria (IPSB). These lower abundances were possibly caused by increased AP in the rhizosphere soil (Figure 4). Previously, Hu et al. (2009) also found that the metabolic activities of IPSB were limited by high P input. Moreover, root exudates and microorganisms were also recognized as the main sources of rhizosphere soil enzymes (Lian et al., 2019). Hence, our findings provide evidence for the competitive exchange of Si and P at the soil microorganism level.

### Effect of Si on Bacterial Association Network

In general, the interactions (i.e., positive, negative, and neutral) between microorganisms in various habitats were explored by association network analysis (Fan et al., 2018; Lian et al., 2019). To compare the network complexities that existed in the rhizosphere soils of the Si0 and Si200 treatments and further investigate the bacterial community composition, the association network analysis was performed (Shi et al., 2016). Multiple network topological metrics consistently indicated that the bacterial networks of Si200 markedly differed from Si0 (Table 4, Figure 6). Compared with Si0, the number of edges, number of positive correlations, graph density, *avgCC*, and *avgK* were higher in Si200, which suggests that Si may be beneficial to enriching PGPR for greater network robustness in the rhizosphere (Fan et al., 2018). Besides, the higher *avgK* and number of positive correlations in Si200 indicated that there are more positive ecological interactions between the dominant bacterial genera (Zhang et al., 2018; Lian et al., 2019). Therefore, these results showed that Si enriched the network of rhizosphere bacteria, which, in turn, could contribute to sugarcane growth.

## Conclusion

Overall, the present study revealed that Si application effectively promoted sugarcane growth by rebalancing the absorption and utilization of nutrients (i.e., N, P, K, and Si), and significantly influenced soil enzyme activity and properties, especially ASi, AP, AK, and SAP. Moreover, Si application also changed rhizosphere soil bacterial structures despite making no marked difference in bacterial diversities, while the AP played the most important role in driving the bacterial community structure between the two Si treatments. Furthermore, Si application significantly enhanced the relative abundances of *Proteobacteria* and may activate PGPR, such as *Bryobacter, Cytophaga, Flavobacterium*, and *Flavisolibacter*, and enrich the network of rhizosphere bacteria, which may be beneficial to sugarcane growth. However, further experiments are required to illustrate the role of these microbes in enhancing sugarcane growth through Si fertilizers.

## Data Availability Statement

The datasets presented in this study can be found in online repositories. The names of the repository/repositories and accession number(s) can be found in the article/Supplementary Material.

## Author Contributions

WS conceived and designed the experimental plan. QD, TY, ZZ, and QS performed the experiments. QD, SH, and UA analyzed the data and wrote the manuscript. WS, UA, TL, JC, and WM revised the paper. All authors have read and approved the final version of the paper.

## Conflict of Interest

The authors declare that the research was conducted in the absence of any commercial or financial relationships that could be construed as a potential conflict of interest.

## Publisher's Note

All claims expressed in this article are solely those of the authors and do not necessarily represent those of their affiliated organizations, or those of the publisher, the editors and the reviewers. Any product that may be evaluated in this article, or claim that may be made by its manufacturer, is not guaranteed or endorsed by the publisher.
