# Supplementary material for: Silicon Application Modulates the Growth, Rhizosphere Soil Characteristics, and Bacterial Community Structure in Sugarcane
Source: Front Plant Sci. 2021 Aug 20;12:710139. doi: 10.3389/fpls.2021.710139 (PMC8417577; doi:10.3389/fpls.2021.710139)
Supplement: Supplementary file 1 [file Data_Sheet_1.docx]

**Silicon application modulates the growth, rhizosphere soil characteristics and bacterial community structure in sugarcane**

*Quanqing Deng^1,2,†^, Taobing Yu^3,†^, Zhen Zeng^1,2,†^, Umair Ashraf^4,†^, Qihan Shi^3^, Suihua Huang^1,3,5^, Tengxiang Lian^3^, Jianwen Chen^1,2^, Wardah Muzaffar^6^, Wankuan Shen^1,2,5*^*

^1^ *College of Agriculture, South China Agricultural University, Guangdong Guangzhou 510642, China*

^2^ *Sugarcane Research Laboratory, South China Agricultural University, Guangzhou, China*

^3^ *The State Key Laboratory for Conservation and Utilization of Subtropical Agro-bioresources, South China Agricultural University, Guangzhou, Guangdong, China*

^4^ *Division of Science and Technology, Department of Botany, University of Education, Lahore, 54770, Punjab, Pakistan*

^5^ *Scientific Observing and Experimental Station of Crop Cultivation in South China, Ministry of Agriculture, Guangdong Guangzhou 510642, China*

^6^ *Sugarcane Research Institute, Ayub Agricultural Research Institute, Faisalabad, Pakistan*

*Corresponding authors: Dr. Prof. Wankuan Shen

Mailing address: College of Agriculture, South China Agricultural University, Guangzhou 510642, China

Tel: +86-20-85280206

Fax: +86-20-85280203

E-mail: wkshen69@126.com

^†^ These authors contributed equally to this work.

**Supplementary material**

**Table S1** Nutritional elements contents (%) of three sugarcane varieties (A, B and C) under two Si treatments

| V | T | Si content | | | N content | | | P content | | | K content | | |
| --- | --- | --- | --- | --- | --- | --- | --- | --- | --- | --- | --- | --- | --- |
|  |  | Leaf | Stalk | Root | Leaf | Stalk | Root | Leaf | Stalk | Root | Leaf | Stalk | Root |
| A | Si0 | 1.20±0.01b | 0.79±0.01d | 1.10±0.01c | 1.55±0.16bc | 1.97±0.14ab | 1.04±0.02a | 0.33±0.01a | 0.409±0.005b | 0.180±0.001c | 1.22±0.01a | 3.79±0.02b | 0.269±0.003cd |
|  | Si200 | 1.31±0.02a | 1.02±0.01b | 1.13±0.01b | 2.48±0.23a | 2.18±0.08a | 0.90±0.07b | 0.30±0.04b | 0.438±0.004a | 0.196±0.001a | 1.11±0.02bc | 2.99±0.01c | 0.356±0.006a |
| B | Si0 | 0.88±0.03e | 0.93±0.01cd | 1.01±0.01d | 1.33±0.26bc | 1.72±0.14ab | 0.84±0.07b | 0.24±0.03d | 0.371±0.002c | 0.161±0.001f | 1.16±0.01b | 3.16±0.04c | 0.213±0.006e |
|  | Si200 | 0.96±0.05d | 0.97±0.02c | 1.34±0.01a | 1.42±0.21bc | 1.83±0.25ab | 0.86±0.05b | 0.22±0.01d | 0.423±0.004ab | 0.165±0.001e | 1.06±0.02d | 2.87±0.03c | 0.243±0.002d |
| C | Si0 | 1.08±0.01c | 0.93±0.01cd | 0.98±0.01e | 1.04±0.16c | 1.67±0.24b | 1.06±0.02a | 0.27±0.09c | 0.322±0.003d | 0.178±0.001d | 1.08±0.01c | 4.08±0.03a | 0.278±0.006bc |
|  | Si200 | 1.10±0.01c | 1.08±0.01a | 1.10±0.02c | 1.65±0.17b | 1.78±0.14ab | 0.82±0.01b | 0.23±0.07d | 0.335±0.006d | 0.182±0.001b | 1.02±0.01d | 3.75±0.18b | 0.303±0.001b |
| ANOVA | V | ** | ns | ns | * | ns | ns | ** | ** | ** | ** | ** | ** |
|  | T | ns | ** | ** | * | ns | * | ns | ns | ns | ** | * | * |
|  | V×T | ** | ** | ** | ** | ns | * | ** | ** | ** | ** | ** | ** |

Note: Different lowercase letters within a column indicate a significant difference under each treatments according to the LSD (0.05). The error term is SE. ns, not significant at the 0.05 probability level; * and ** , significant at the 0.05 and 0.01 probability levels, respectively. V, variety. T, treatment.

**Table S2** Soil physicochemical properties of three sugarcane varieties (A, B and C) under two Si treatments

| V | T | pH | ASi  (mg kg^-1^) | AN  (mg kg^-1^) | AP  (mg kg^-1^) | AK  (mg kg^-1^) | SOM  (%) | TN  (%) | TP  (%) | TK  (%) |
| --- | --- | --- | --- | --- | --- | --- | --- | --- | --- | --- |
| A | Si0 | 5.37±0.03b | 76.66±1.67b | 53.86±0.34bc | 28.33±0.10c | 15.70±0.15d | 2.16±0.08a | 0.084±0.002b | 0.217±0.001bc | 2.072±0.007ab |
|  | Si200 | 5.66±0.04a | 88.29±1.69a | 54.97±0.35a | 34.77±0.22a | 21.97±0.27a | 2.18±0.09a | 0.093±0.001a | 0.228±0.001a | 2.078±0.003a |
| B | Si0 | 5.25±0.05c | 56.66±2.20c | 53.91±0.01bc | 28.59±0.30c | 17.54±0.35c | 2.06±0.08a | 0.084±0.001b | 0.216±0.001cd | 2.030±0.015bc |
|  | Si200 | 5.41±0.02b | 71.64±0.84b | 53.65±0.18c | 31.36±0.74b | 21.81±0.22a | 2.11±0.09ab | 0.088±0.002ab | 0.227±0.001a | 2.060±0.004b |
| C | Si0 | 5.44±0.02b | 52.44±1.44d | 54.51±0.32ab | 28.08±0.19c | 20.31±0.19b | 1.89±0.08a | 0.084±0.003b | 0.214±0.001d | 2.023±0.026c |
|  | Si200 | 5.61±0.03a | 91.65±0.83a | 53.91±0.01bc | 36.35±0.82a | 22.06±0.23a | 2.12±0.08c | 0.085±0.002b | 0.219±0.001b | 2.075±0.013ab |
| ANOVA | V | * | * | ns | ns | ns | * | * | ns | ns |
|  | T | ** | ** | ns | ** | ** | * | * | ** | * |
|  | V×T | ** | ** | * | ** | ** | ** | ** | ** | * |

Note: Different lowercase letters within a column indicate a significant difference under each treatments according to the LSD (0.05). The error term is SE. ns, not significant at the 0.05 probability level; * and ** , significant at the 0.05 and 0.01 probability levels, respectively. V, variety. T, treatment.

**Table S3** Rhizosphere soil diversity indices of three sugarcane varieties (A, B and C) under two Si treatments

| V | T | Coverage % | OTUs | Chao1 | Shannon_e |
| --- | --- | --- | --- | --- | --- |
| A | Si0 | 98.07±0.09a | 4713.00±121.69a | 4724.13±118.26a | 6.49±0.07a |
|  | Si200 | 98.07±0.03a | 4373.67±334.23a | 4380.17±335.09a | 6.54±0.10a |
| B | Si0 | 98.07±0.09a | 4750.00±193.34a | 4757.77±191.14a | 6.59±0.06a |
|  | Si200 | 98.07±0.03a | 5195.33±85.21a | 5203.87±83.27a | 6.93±0.05a |
| C | Si0 | 98.13±0.03a | 4827.67±170.64a | 4833.33±172.27a | 6.66±0.03a |
|  | Si200 | 98.10±0.01a | 4744.67±360.05a | 4754.60±357.31a | 6.18±0.39a |
| ANOVA | V | ns | ns | ns | ns |
|  | T | ns | ns | ns | ns |
|  | V×T | ns | ns | ns | ns |

Note: Different lowercase letters within a column indicate a significant difference under each treatments according to the Sidak-Holm (0.05). The error term is SE. ns, not significant at the 0.05 probability level.

**Table S4** The Pearson's correlation coefficients between the OTUs (only enriched in the Si200) and Si treatments

| OTUID | Correlation | Kingdom | Phylumn | Class | Order | Family | Genus | Species |
| --- | --- | --- | --- | --- | --- | --- | --- | --- |
| OTU1477 | 0.48* | Bacteria | Bacteroidetes | Sphingobacteriia | Sphingobacteriales | PHOS-HE51 | |  |
| OTU1734 | 0.48* | Bacteria | Verrucomicrobia | Verrucomicrobiae | Verrucomicrobiales | Verrucomicrobiaceae | Luteolibacter | |
| OTU1763 | 0.48* | Bacteria | Chlamydiae | Chlamydiae | Chlamydiales | Simkaniaceae | Candidatus_Rhabdochlamydia | |
| OTU1478 | 0.49* | Bacteria | Bacteroidetes | Sphingobacteriia | Sphingobacteriales | env.OPS_17 | |  |
| OTU4956 | 0.49* | Bacteria | Bacteroidetes | Sphingobacteriia | Sphingobacteriales | Chitinophagaceae | Flavisolibacter | Flavisolibacter_sp._LY43 |
| OTU2236 | 0.50* | Bacteria |  |  |  |  |  |  |
| OTU5450 | 0.50* | Bacteria | Firmicutes | OPB54 |  |  |  |  |
| OTU4687 | 0.50* | Bacteria | Bacteroidetes | Sphingobacteriia | Sphingobacteriales | env.OPS_17 | |  |
| OTU12151 | 0.52* | Bacteria | Proteobacteria | Betaproteobacteria | Rhodocyclales | Rhodocyclaceae | |  |
| OTU3919 | 0.53* | Bacteria | Proteobacteria | Alphaproteobacteria | Rickettsiales | TK34 |  |  |
| OTU3439 | 0.53* | Bacteria | Chloroflexi | Anaerolineae | Anaerolineales | Anaerolineaceae | |  |
| OTU2347 | 0.53* | Bacteria | Bacteroidetes | Sphingobacteriia | Sphingobacteriales | Sphingobacteriaceae | Arcticibacter | |
| OTU2112 | 0.53* | Bacteria | Thermotogae | Thermotogae | Thermotogales | Thermotogaceae | GAL15 |  |
| OTU1658 | 0.54* | Bacteria | Proteobacteria | Alphaproteobacteria | Rhizobiales | Rhodobiaceae | |  |
| OTU2470 | 0.54* | Bacteria | Chloroflexi | TK10 |  |  |  |  |
| OTU5155 | 0.55* | Bacteria | Planctomycetes | Phycisphaerae | WD2101_soil_group | |  |  |
| OTU15094 | 0.55* | Bacteria | Chloroflexi | Anaerolineae | Anaerolineales | Anaerolineaceae | |  |
| OTU1864 | 0.56* | Bacteria | Proteobacteria | Betaproteobacteria | Burkholderiales | Comamonadaceae | |  |
| OTU2272 | 0.56* | Bacteria | Gemmatimonadetes | Gemmatimonadetes | Gemmatimonadales | Gemmatimonadaceae | | |
| OTU5701 | 0.57* | Bacteria | Proteobacteria | Betaproteobacteria | Burkholderiales | Alcaligenaceae | |  |
| OTU6081 | 0.57* | Bacteria | Chloroflexi | Anaerolineae | Anaerolineales | Anaerolineaceae | |  |
| OTU2839 | 0.58* | Bacteria | Proteobacteria | Gammaproteobacteria | Chromatiales | Ectothiorhodospiraceae | Acidiferrobacter | |
| OTU1542 | 0.58* | Bacteria | Omnitrophica | NPL-UPA2 | |  |  |  |
| OTU975 | 0.59* | Bacteria | Acidobacteria | Acidobacteria | Subgroup_3 | Unknown_Family | Bryobacte | Acidobacteriaceae_bacterium_LX51 |
| OTU2098 | 0.60** | Bacteria | Proteobacteria | Deltaproteobacteria | Desulfovibrionales | Desulfovibrionaceae | Desulfovibrio | |
| OTU1531 | 0.61** | Bacteria | Actinobacteria | Actinobacteria | Streptosporangiales | Nocardiopsaceae | Nocardiopsis | |
| OTU1523 | 0.64** | Bacteria | Planctomycetes | Planctomycetacia | Planctomycetales | Planctomycetaceae | |  |
| OTU1975 | 0.65** | Bacteria | Planctomycetes | Phycisphaerae | WD2101_soil_group | |  |  |
| OTU3504 | 0.66** | Bacteria | Bacteroidetes | Flavobacteriia | Flavobacteriales | Flavobacteriaceae | Flavobacterium | Flavobacterium_sp._THWCSN34 |
| OTU3634 | 0.67** | Bacteria | Bacteroidetes | Cytophagia | Cytophagales | Cytophagaceae | Cytophaga | |
| OTU2602 | 0.68** | Bacteria | Proteobacteria | Alphaproteobacteria | Rhodospirillales | DA111 |  |  |
| OTU5051 | 0.68** | Bacteria | Proteobacteria | Deltaproteobacteria | Myxococcales | Sandaracinaceae | |  |
| OTU852 | 0.68** | Bacteria | Parcubacteria | |  |  |  |  |
| OTU2504 | 0.69** | Bacteria | Spirochaetae | Spirochaetes | Spirochaetales | Spirochaetaceae | |  |
| OTU1245 | 0.70** | Bacteria | Parcubacteria | |  |  |  |  |
| OTU9693 | 0.71** | Bacteria | Verrucomicrobia | OPB35_soil_group | |  |  |  |
| OTU2333 | 0.71** | Bacteria | Chloroflexi | Caldilineae | Caldilineales | Caldilineaceae | |  |
| OTU4792 | 0.71** | Bacteria | Firmicutes | Clostridia | Clostridiales | Ruminococcaceae | |  |
| OTU2313 | 0.72** | Bacteria | Proteobacteria | Deltaproteobacteria | Sh765B-TzT-29 | |  |  |

**Table S5** The Spearman's correlations between the rhizosphere soil properties and bacterial community structures (Bray-Curtis distance) as determined by Mantel test

|  | *r*(A) | *P*(A) | *r*(B) | *P*(B) | *r*(C) | *P*(C) | *r*(Si) | *P*(Si) |
| --- | --- | --- | --- | --- | --- | --- | --- | --- |
| pH | 0.23 | 0.146 | 0.67 | **0.008** | 0.07 | 0.401 | 0.03 | 0.353 |
| ASi | 0.52 | **0.001** | 0.83 | 0.062 | 0.08 | 0.218 | 0.08 | 0.220 |
| SOM | -0.08 | 0.554 | -0.09 | 0.436 | -0.12 | 0.367 | -0.09 | 0.694 |
| TN | 0.05 | 0.338 | -0.06 | 0.553 | -0.25 | 0.912 | -0.05 | 0.609 |
| TP | 0.23 | 0.097 | 0.91 | 0.086 | 0.04 | 0.274 | 0.08 | 0.244 |
| TK | 0.25 | 0.233 | 0.3 | 0.174 | -0.09 | 0.335 | -0.1 | 0.645 |
| AN | 0.35 | 0.97 | 0.25 | 0.264 | -0.19 | 0.597 | 0.03 | 0.324 |
| AP | 0.34 | **0.028** | 0.47 | 0.092 | 0.34 | **0.035** | 0.47 | **0.001** |
| AK | 0.26 | 0.092 | 0.93 | 0.053 | 0.21 | 0.118 | 0.1 | 0.119 |
| SCAT | 0.37 | 0.101 | 0.18 | 0.251 | 0.33 | 0.269 | -0.02 | 0.402 |
| SAP | 0.29 | 0.058 | 0.95 | **0.035** | 0.11 | 0.203 | -0.06 | 0.554 |
| SI | 0.88 | **0.013** | -0.3 | 0.974 | 0.89 | **0.001** | 0.27 | 0.096 |

Note: Bold font indicates significant at the 0.05 or 0.01 probability level


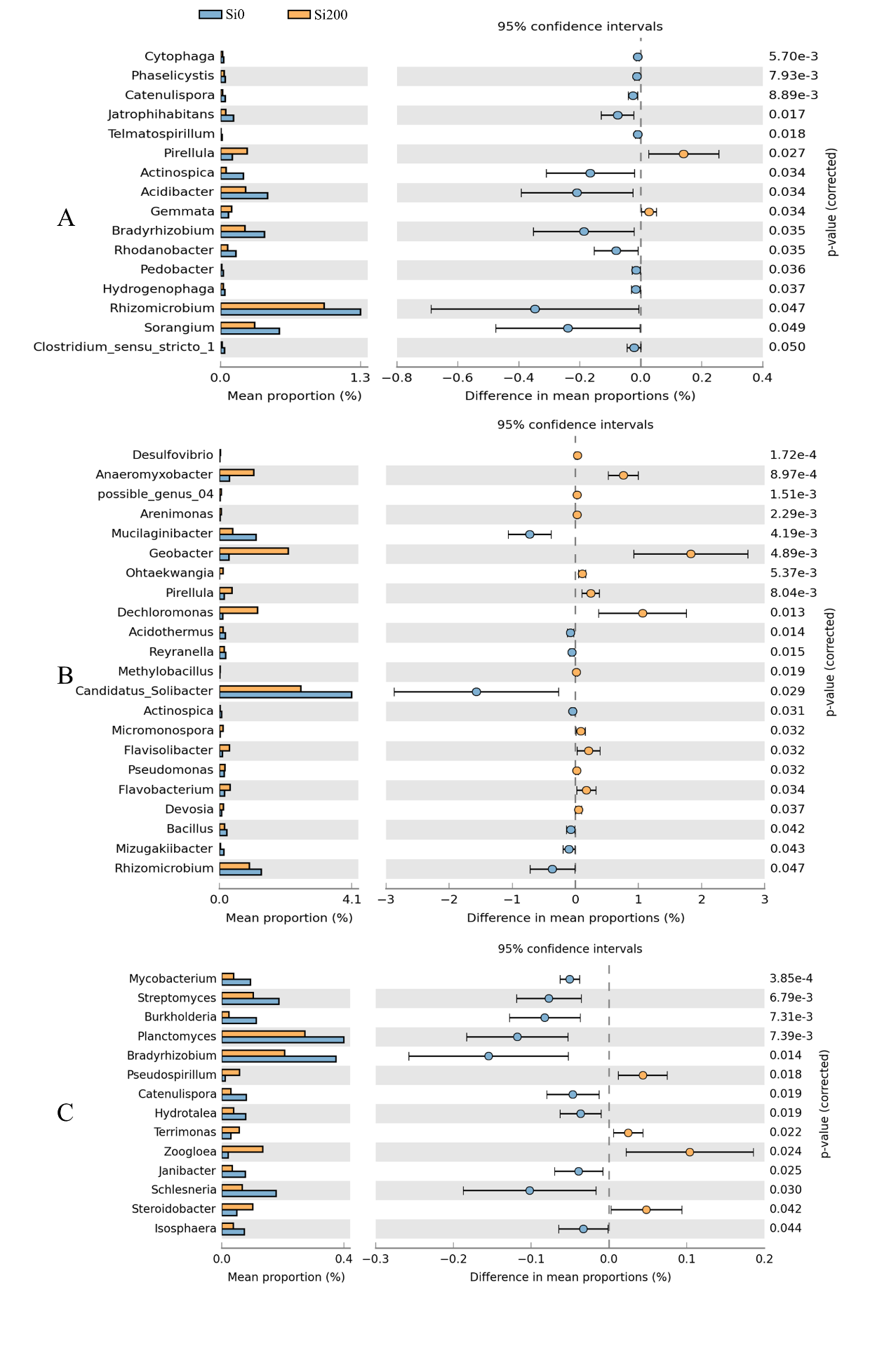


Figure S1 Relative abundance of the genera of the three varieties (A, B and C) under two Si treatments. The error bars show the calculated standard variation of two treatments. Corrected P-values were calculated using the Storey false discovery rate approach (P <0.05)


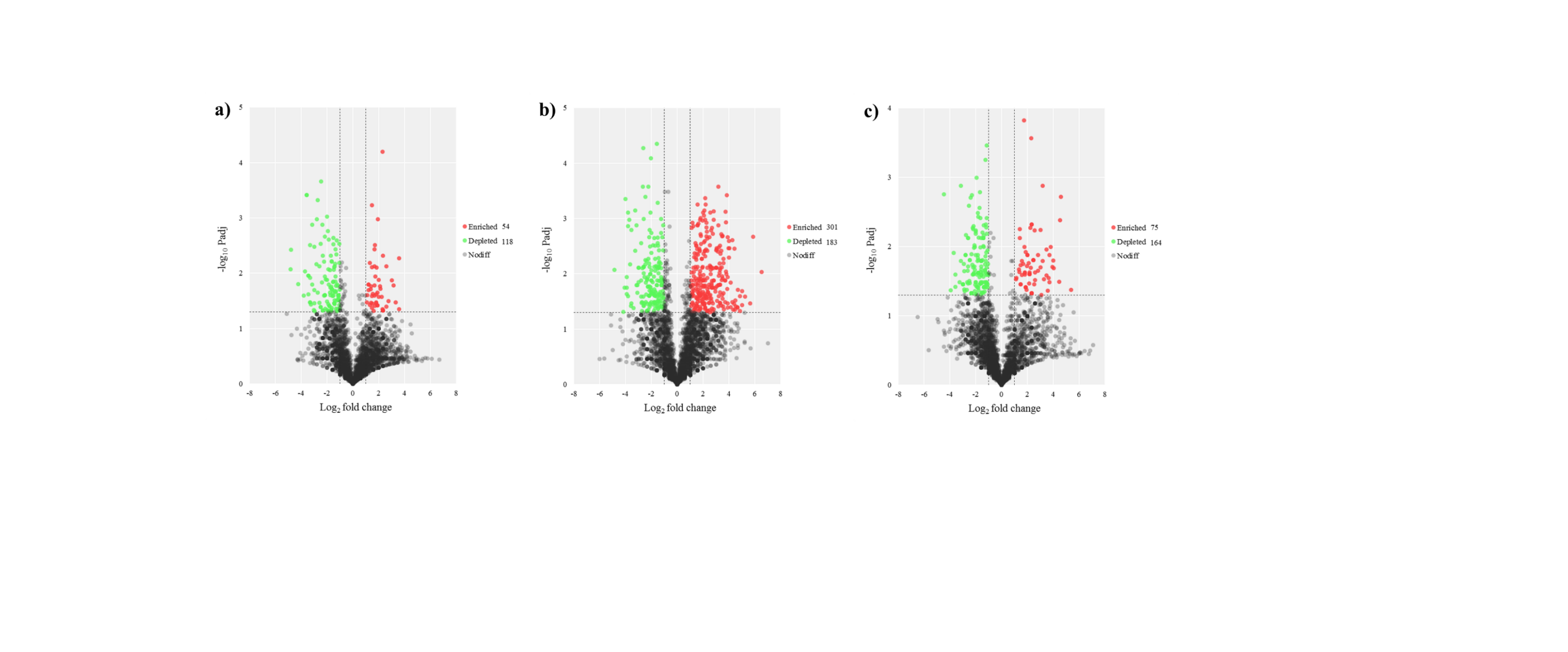


Figure S2 Enrichment and depletion of OTUs of VA (**a**), VB (**b**), and VC (**c**) included in Si200 compared with Si0 as determined by differential abundance analysis. Each point represents an individual OTU, and the position along the y-axis represents the abundance fold-change compared with Si0 (**a**, **b**, **c**)


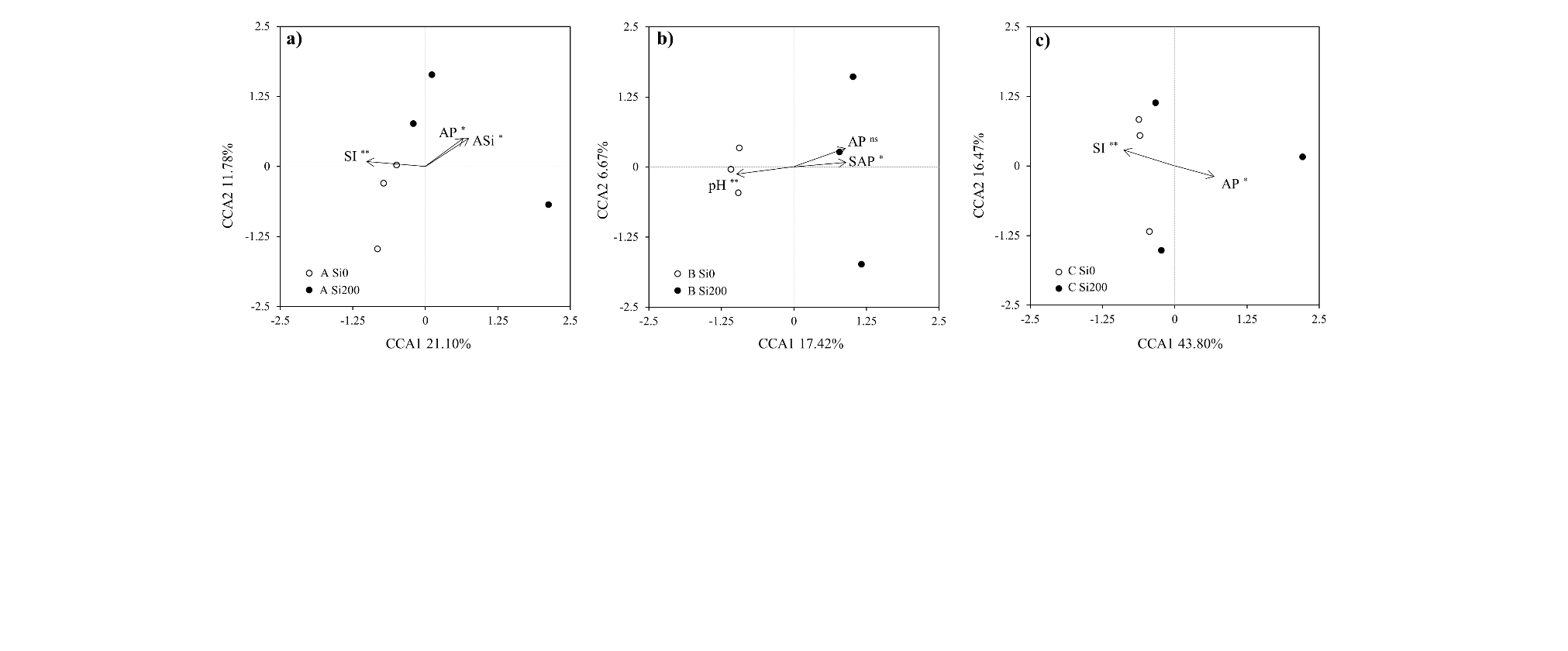


Figure S3 Canonical correspondence analysis (CCA) based on the bacterial community compositions of VA (a), VB (b), VC (c) under two Si treatments. ns, not significant at the 0.05 probability level; * and **, significant at the 0.05 and 0.01 probability levels, respectively.
